# Supplementary material for: Para-Substituted O-Benzyl Sulfohydroxamic Acid Derivatives as Redox-Triggered Nitroxyl (HNO) Sources
Source: Molecules. 2022 Aug 19;27(16):5305. doi: 10.3390/molecules27165305 (PMC9414458; doi:10.3390/molecules27165305)
Supplement: Supplementary file 1 [file molecules-27-05305-s001.zip › molecules-1849075-supplementary.pdf]

Supporting Information

Para Substituted *O*-Benzyl Sulfohydroxamic Acid Derivatives as Redox Triggered Nitroxyl (HNO) Sources

Yueming Long, Zijun Xia, Allison M. Rice, and S. Bruce King

Table of contents: **page number**

1. Materials	S3
2. Figure S1. ^{1}H NMR and ^{13}C NMR of **1a**	S4
3. Figure S2. ^{1}H NMR and ^{13}C NMR of **1b**	S5
4. Figure S3. ^{1}H NMR and ^{13}C NMR of **2b**	S6
5. Figure S4. IR of **2b**	S7
5. Figure S5. ^{1}H NMR and ^{13}C NMR of **3**	S8
6. Figure S6. ^{1}H NMR of **4**	S9
7. Figure S7. ^{1}H NMR of **5**	S9
8. Figure S8. ^{1}H NMR and ^{13}C NMR of **6**	S10
9. Figure S9. ^{1}H NMR and ^{13}C NMR of **1c**	S11
10. Figure S10. ^{1}H NMR of **7**	S12
11. Figure S11. ^{1}H NMR of **8**	S12
12. Figure S12. ^{1}H NMR and ^{13}C NMR of **1d**	S13
13. Figure S13. ^{1}H NMR and ^{13}C NMR of **2a**	S14
14. Figure S14. ^{1}H NMR and ^{13}C NMR of **2c**	S15
15. Figure S15. IR of **2c**	S16
15. Figure S16. MS of **1a**	S17
16. Figure S17. MS of **1b**	S18
17. Figure S18. MS of **2b**	S19
18. Figure S19. MS of **1c**	S20
19. Figure S20. MS of **1d**	S21
20. Figure S21. MS of **2a**	S22
21. Figure S22. MS of **2c**	S23

Materials

The synthetic starting materials and solvent were purchased from the following vendors and used without further purification. *p*-toluenesulphonyl chloride (98%, Alfa Aesar), methanesulfonyl chloride (>99.5%, Sigma Aldrich)), trifluoromethanesulfonyl chloride (98%, Alfa Aesar), *O*-benzylhydroxylamine hydrochloride (99%, Sigma-Aldrich), 1-[(aminooxy)methyl]-4-nitrobenzene hydrochloride (>95%, Matrix Scientific), pyridine (>99%, ACROS Organics), 4-hydroxymethylphenylboronic acid, *N*-hydroxyphthalimide (97%, Sigma Aldrich), pinacol ester (97%, Combi Blocks), diethyl azodicarboxylate (97%, Alfa Aesar), triphenylphosphine (99%, Sigma Aldrich), *p*-toluidine (>99%, Alfa Aesar), benzene (99%, Beantown Chemical), sodium azide (≥99%, Sigma-Aldrich), sodium nitrite (99.5%, Sigma Aldrich), *N*-bromosuccinimide (99%, Alfa Aesar), 2,2'-azobis(2-methylpropionitrile) (98%, Sigma Aldrich), hydrazine monohydrate (>98%, Alfa Aesar), potassium carbonate (>99%, ACROS Organics), ethyl acetate (Fischer Scientific), methylene chloride (Fischer Scientific), chloroform (Fischer Scientific).

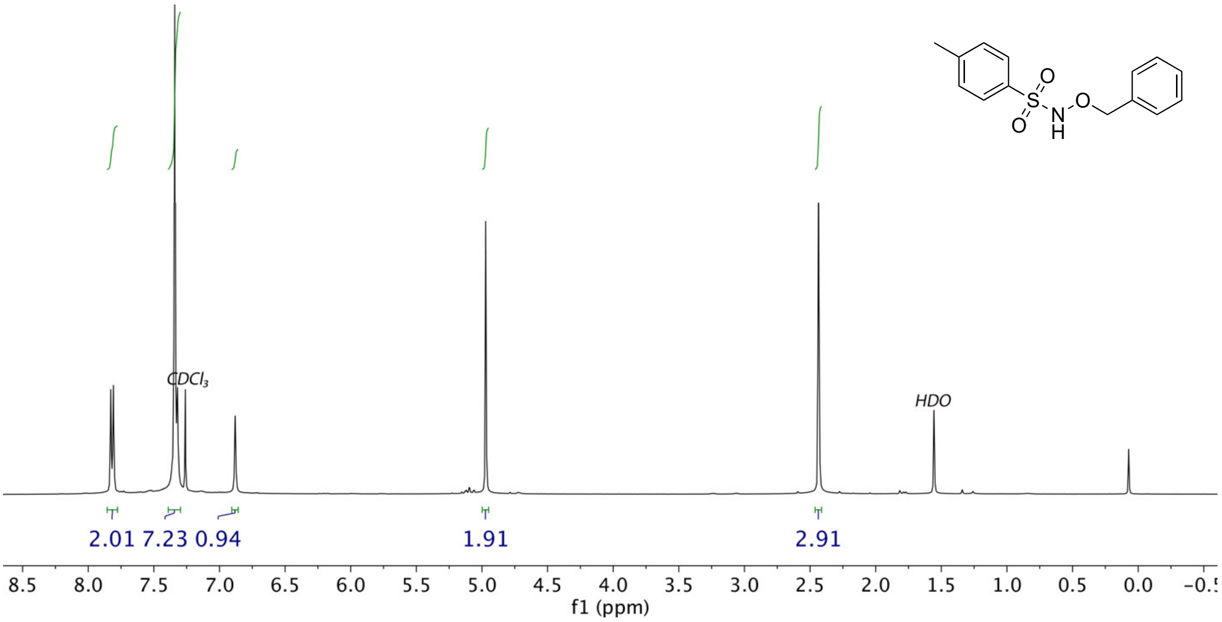

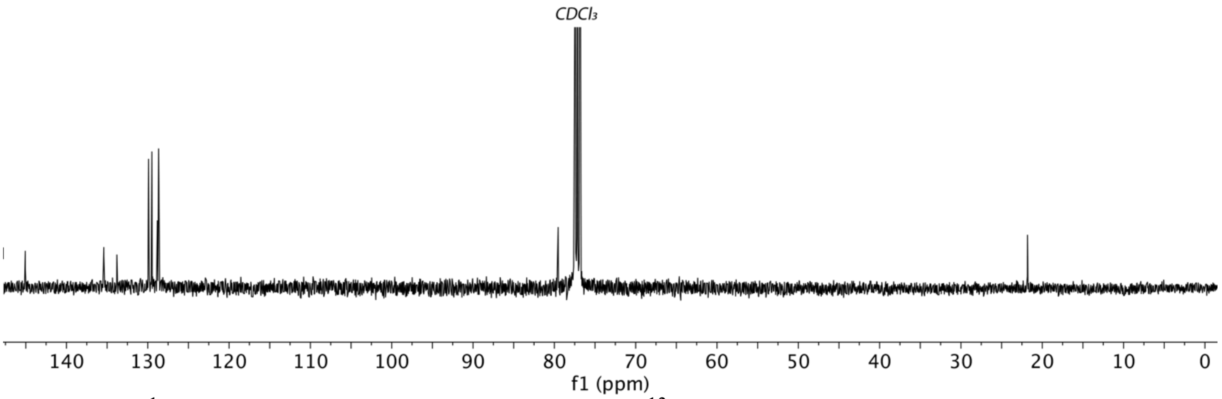

Figure S1. ^{1}H NMR (400 MHz, top) spectrum and ^{13}C NMR (101 MHz, bottom) spectrum of ***N*-(benzyloxy)-4-methylbenzenesulfonamide (1a)** in $CDCl_3$.

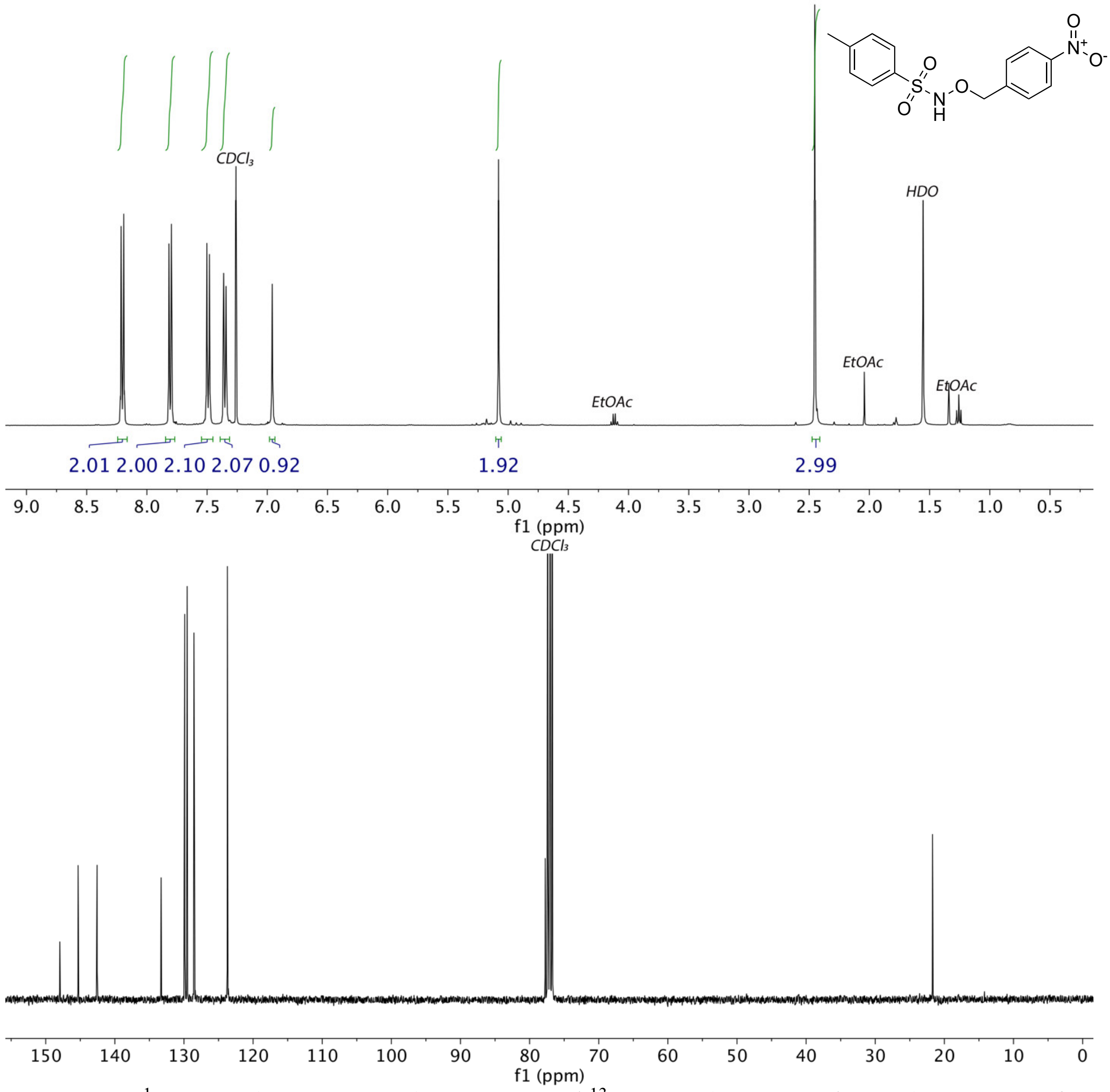

Figure S2. ^{1}H NMR (400 MHz, top) spectrum and ^{13}C NMR (101 MHz, bottom) spectrum of **4-methyl-*N*-((4-nitrobenzyl)oxy)benzenesulfonamide (1b)** in $CDCl_3$.

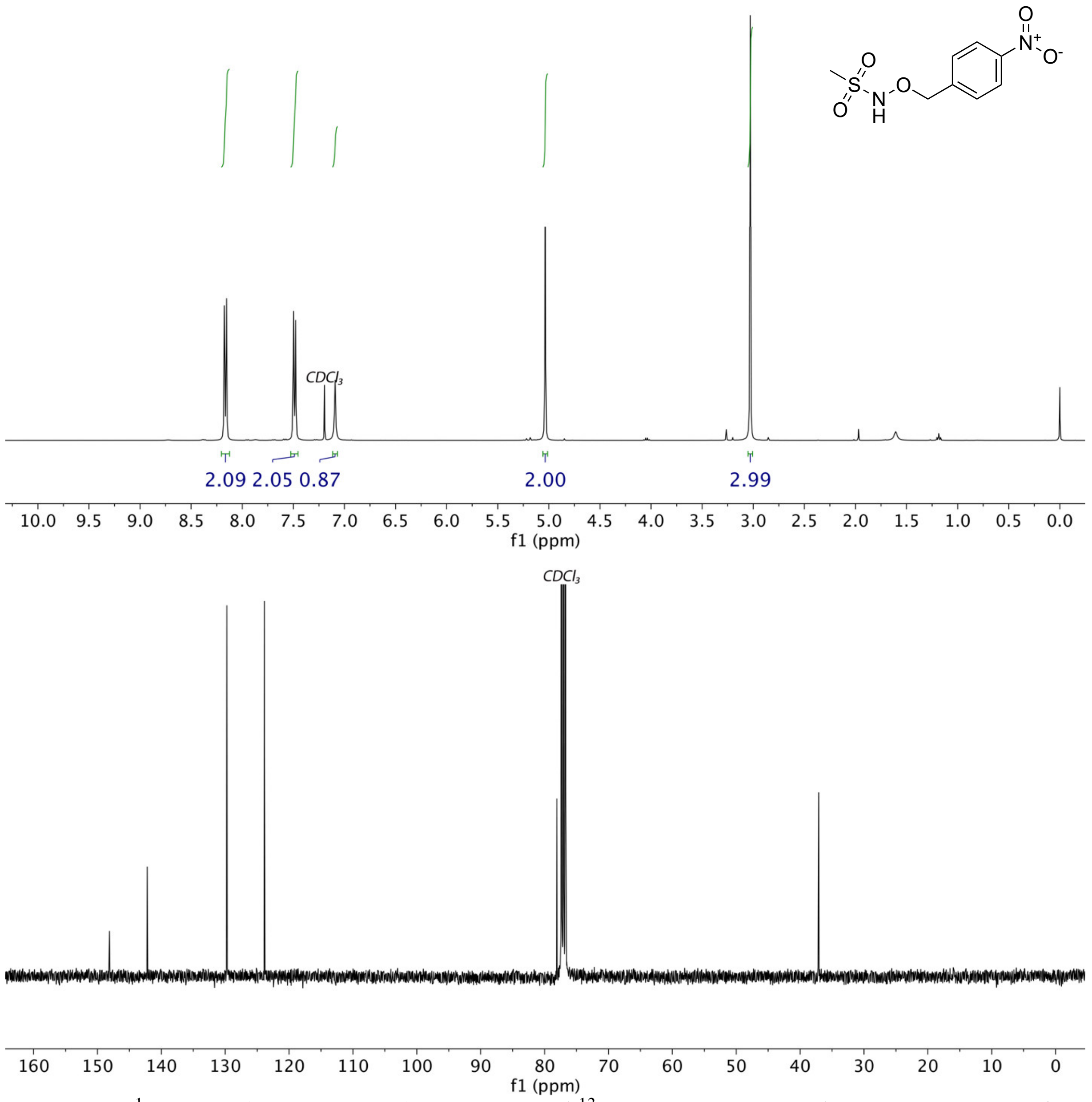

Figure S3. ^{1}H NMR (400 MHz, top) spectrum and ^{13}C NMR (101 MHz, bottom) spectrum of ***N*-((4-nitrobenzyl)oxy)methanesulfonamide (2b)** in $CDCl_3$.

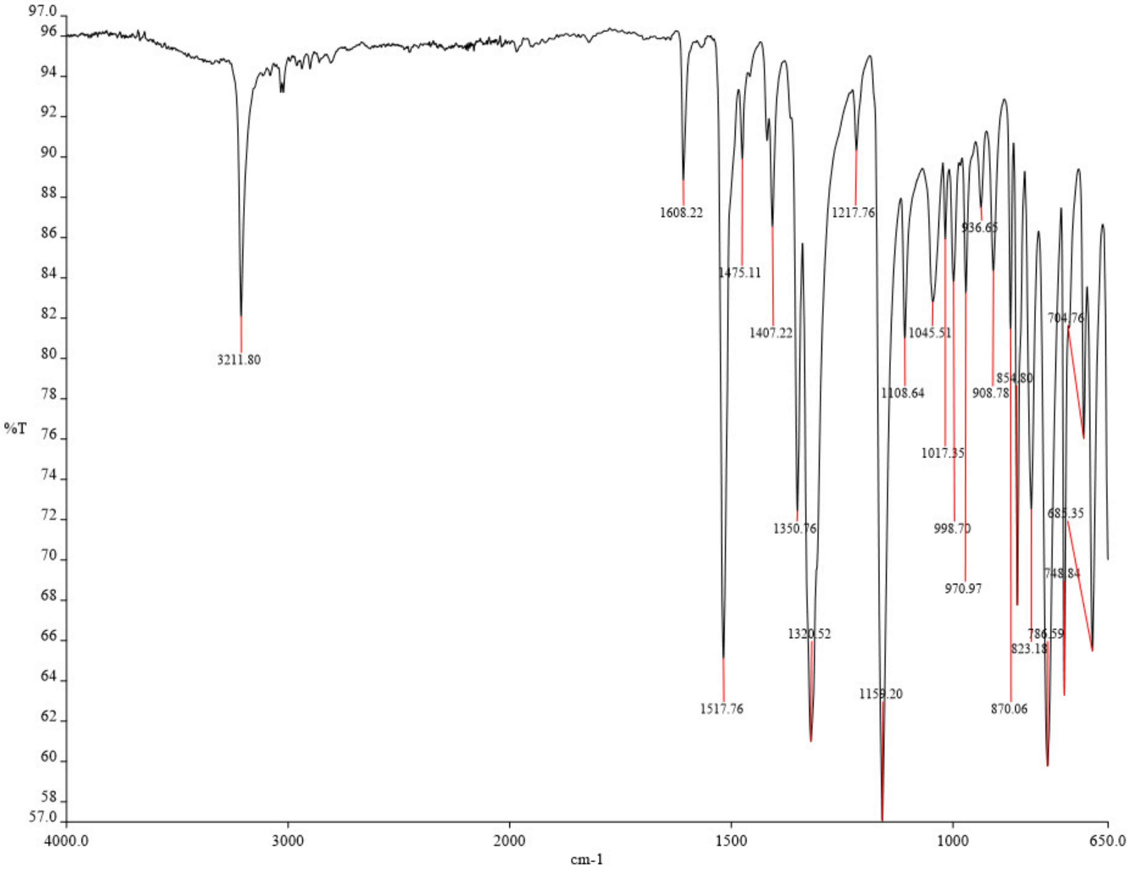

Figure S4. IR of ***N*-((4-nitrobenzyl)oxy)methanesulfonamide (2b)**.

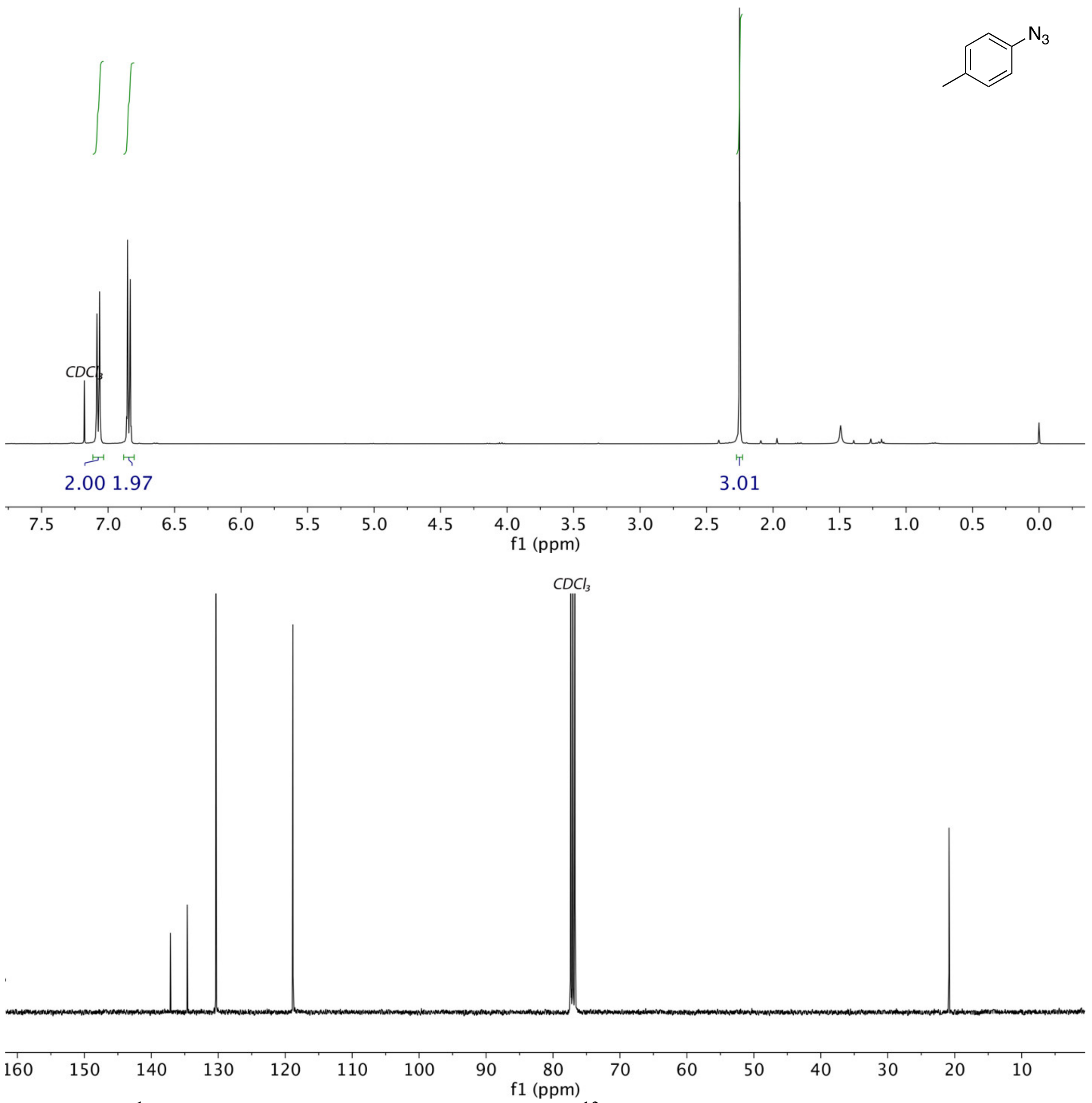

Figure S5. ^{1}H NMR (400 MHz, top) spectrum and ^{13}C NMR (101 MHz, bottom) spectrum of **1-azido-4-methylbenzene (3)** in $CDCl_3$.

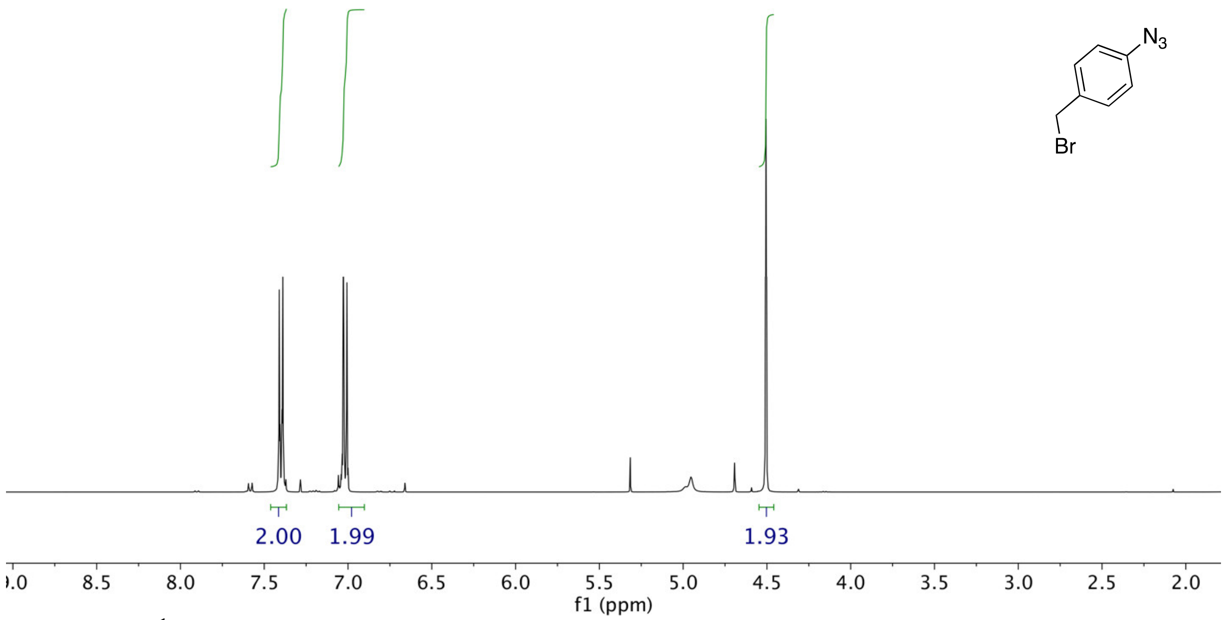

Figure S6. ^{1}H NMR (400 MHz) spectrum of **1-azido-4-(bromomethyl)benzene (4)** in $CDCl_3$.

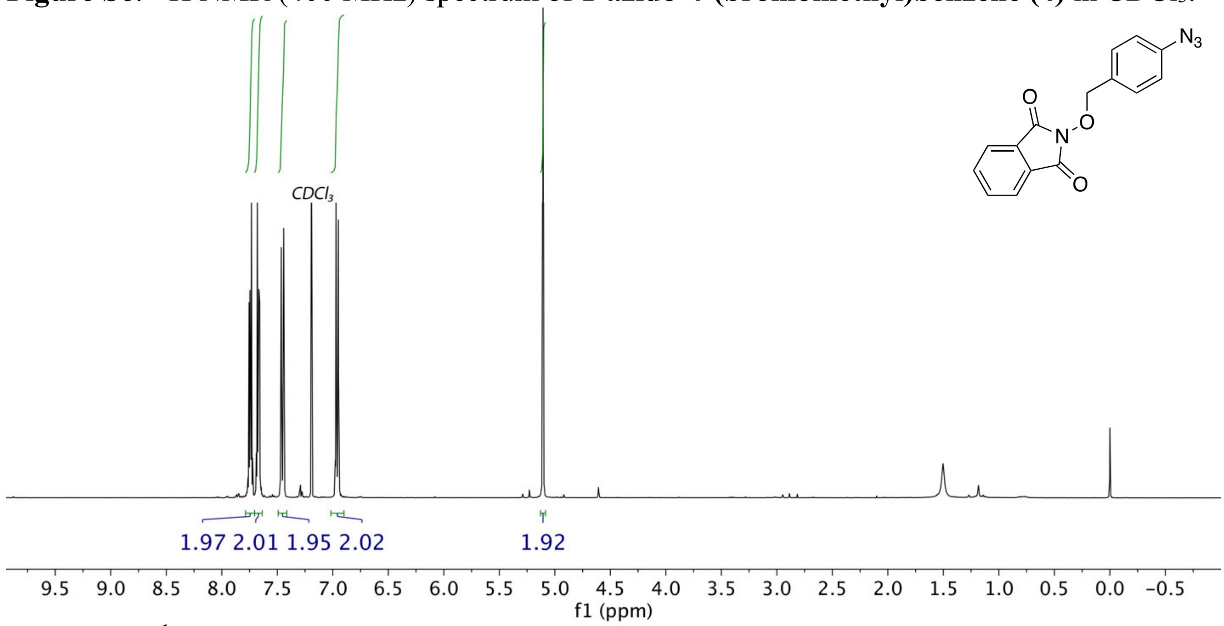

Figure S7. ^{1}H NMR (400 MHz) spectrum of **2-((4-azidobenzyl)oxy)-isoindoline-1,3-dione (5)** in $CDCl_3$.

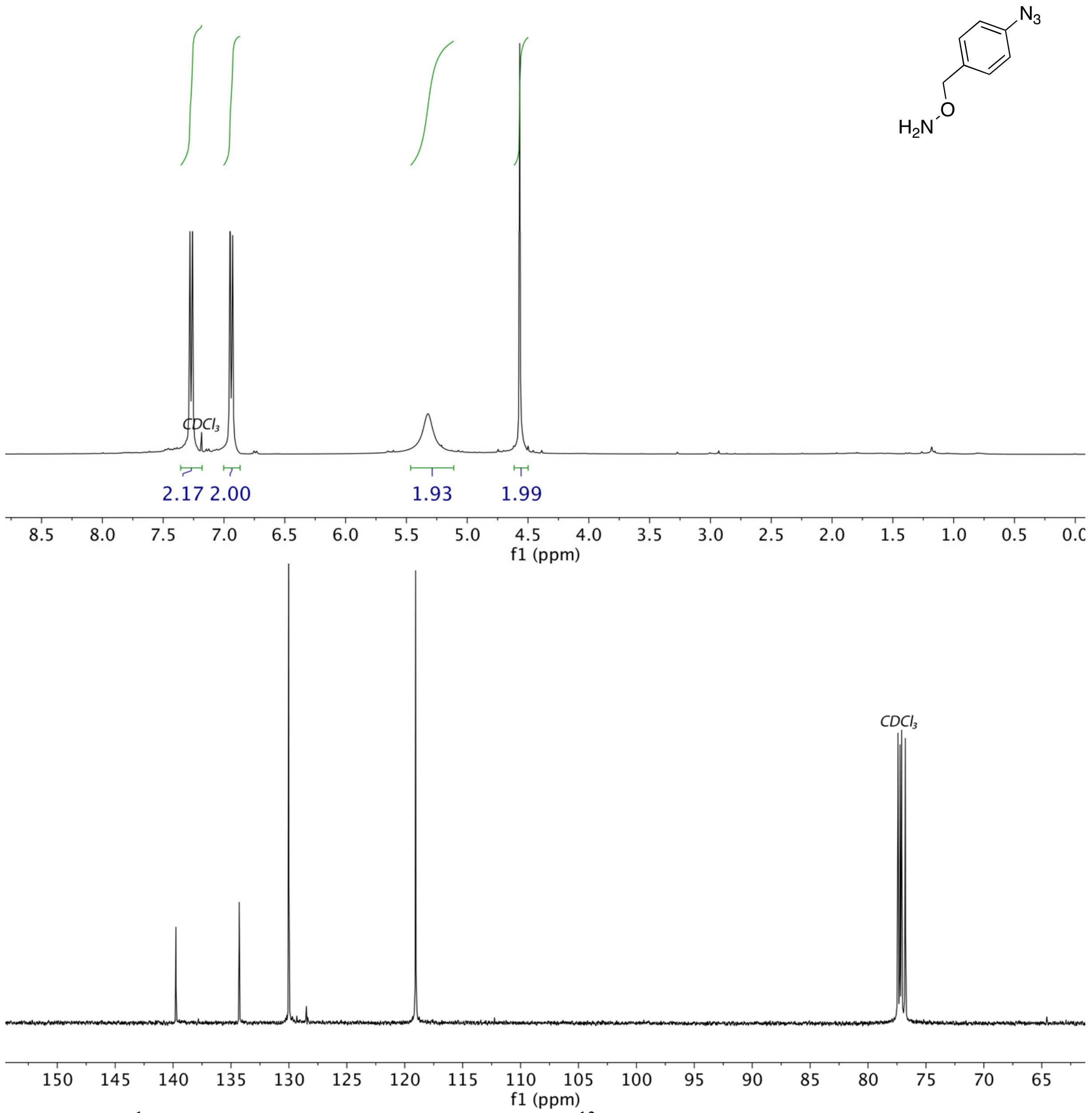

Figure S8. ^{1}H NMR (400 MHz, top) spectrum and ^{13}C NMR (101 MHz, bottom) spectrum of ***O*-(4-azidobenzyl) hydroxylamine (6)** in $CDCl_3$.

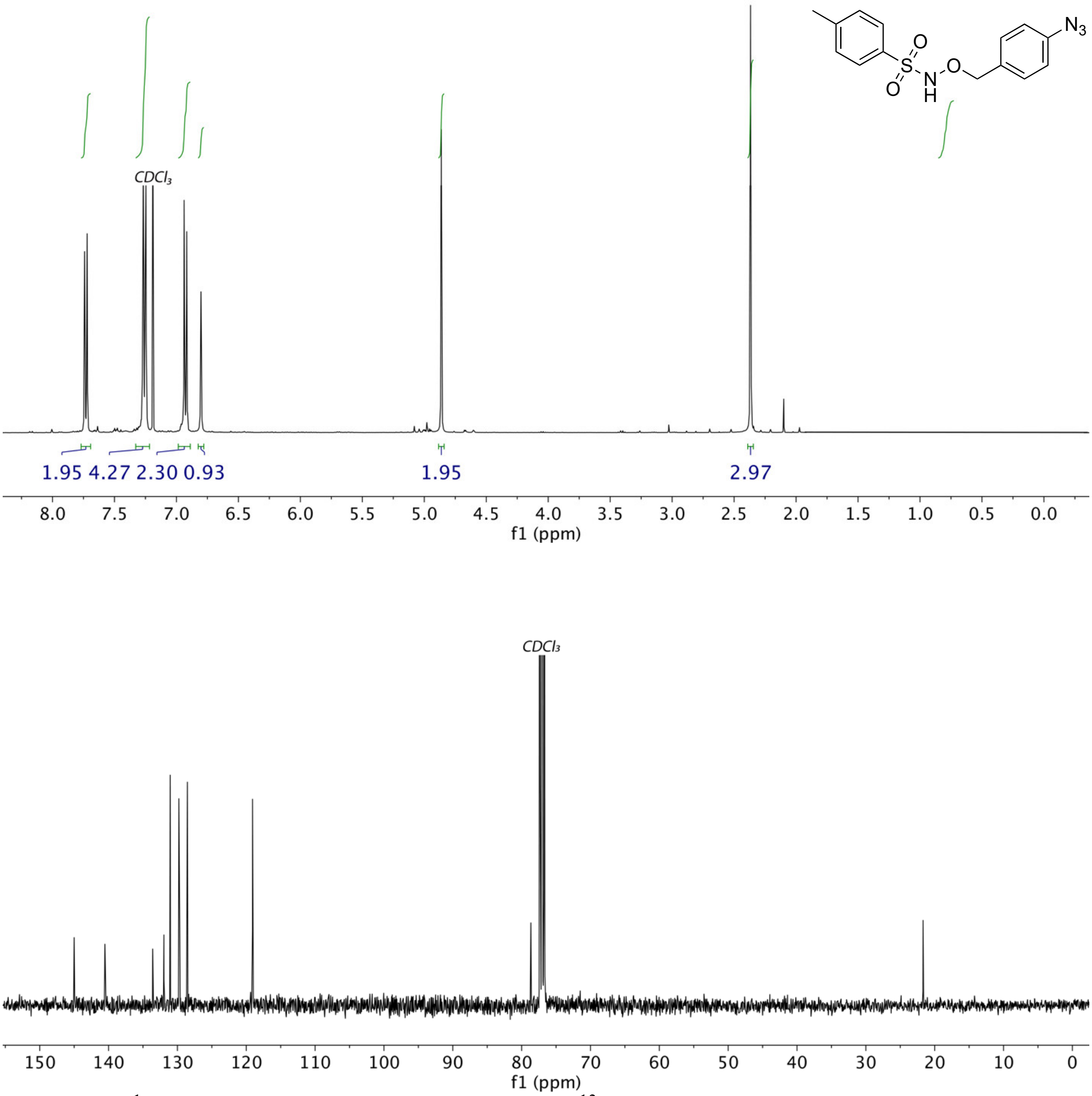

Figure S9. ^{1}H NMR (400 MHz, top) spectrum and ^{13}C NMR (101 MHz, bottom) spectrum of ***N*-((4-azidobenzyl)oxy)-4-methylbenzenesulfonamide (1c)** in $CDCl_3$.

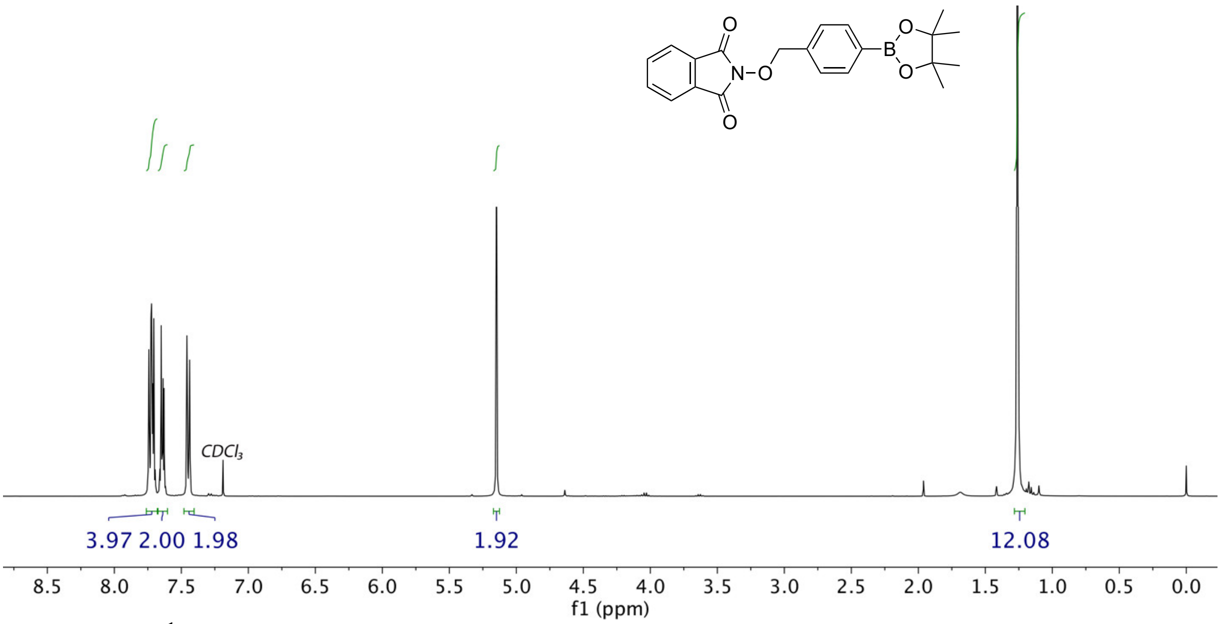

Figure S10. ^{1}H NMR (400 MHz) spectrum of **2-(4-(4,4,5,5-tetramethyl-1,3,2-dioxaborolan-2-yl)phenoxy)isoindoline-1,3-dione (7)** in $CDCl_3$.

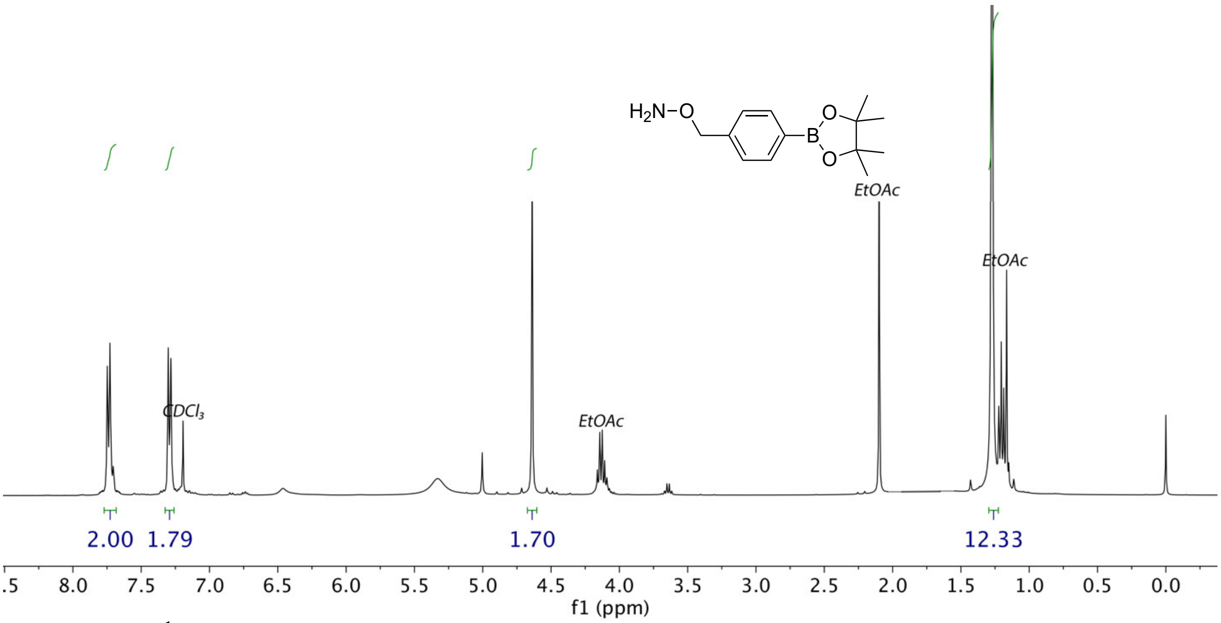

Figure S11. ^{1}H NMR (400 MHz) spectrum of ***O*-(4-(4,4,5,5-tetramethyl-1,3,2-dioxaborolan-2-yl)benzyl)hydroxylamine (8)** in $CDCl_3$.

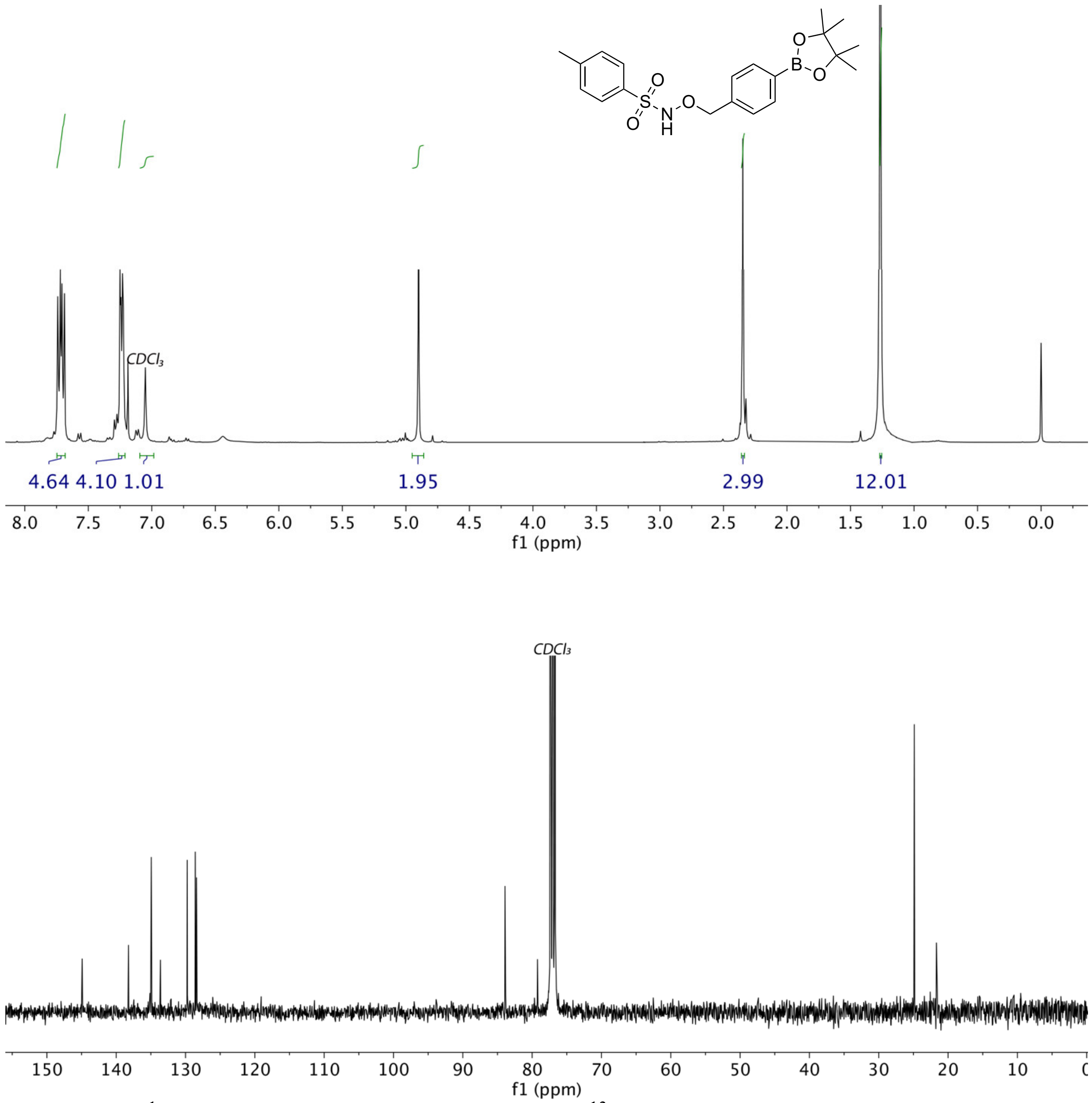

Figure S12. ^{1}H NMR (400 MHz, top) spectrum and ^{13}C NMR (101 MHz, bottom) spectrum of **4-methyl-*N*-((4-(4,4,5,5-tetramethyl-1,3,2-dioxaborolan-2-yl)benzyl)oxy)benzenesulfonamide (1d)** in $CDCl_3$.

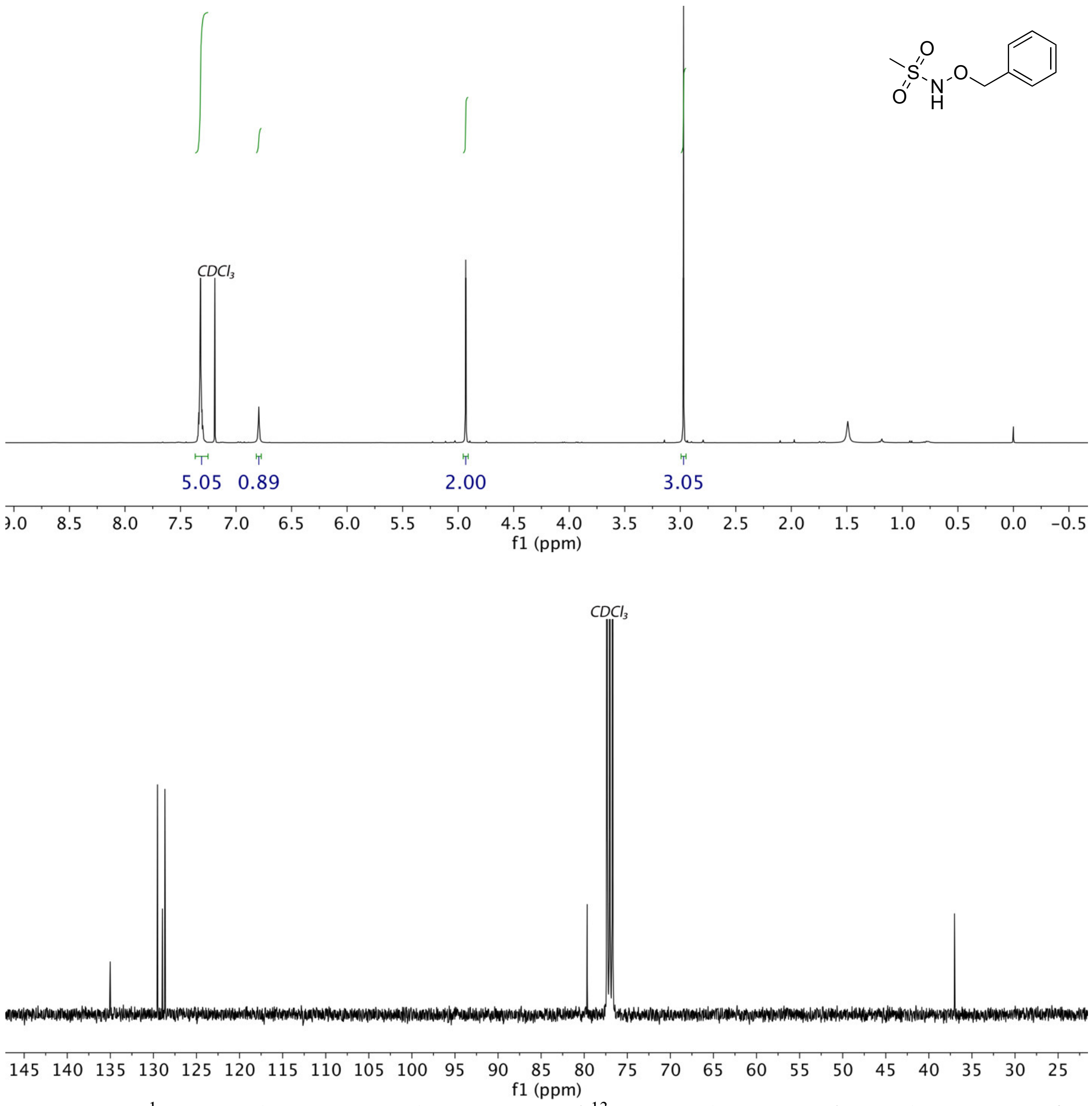

Figure S13. ^{1}H NMR (400 MHz, top) spectrum and ^{13}C NMR (101 MHz, bottom) spectrum of ***N*-(benzyloxy)methanesulfonamide (2a)** in $CDCl_3$.

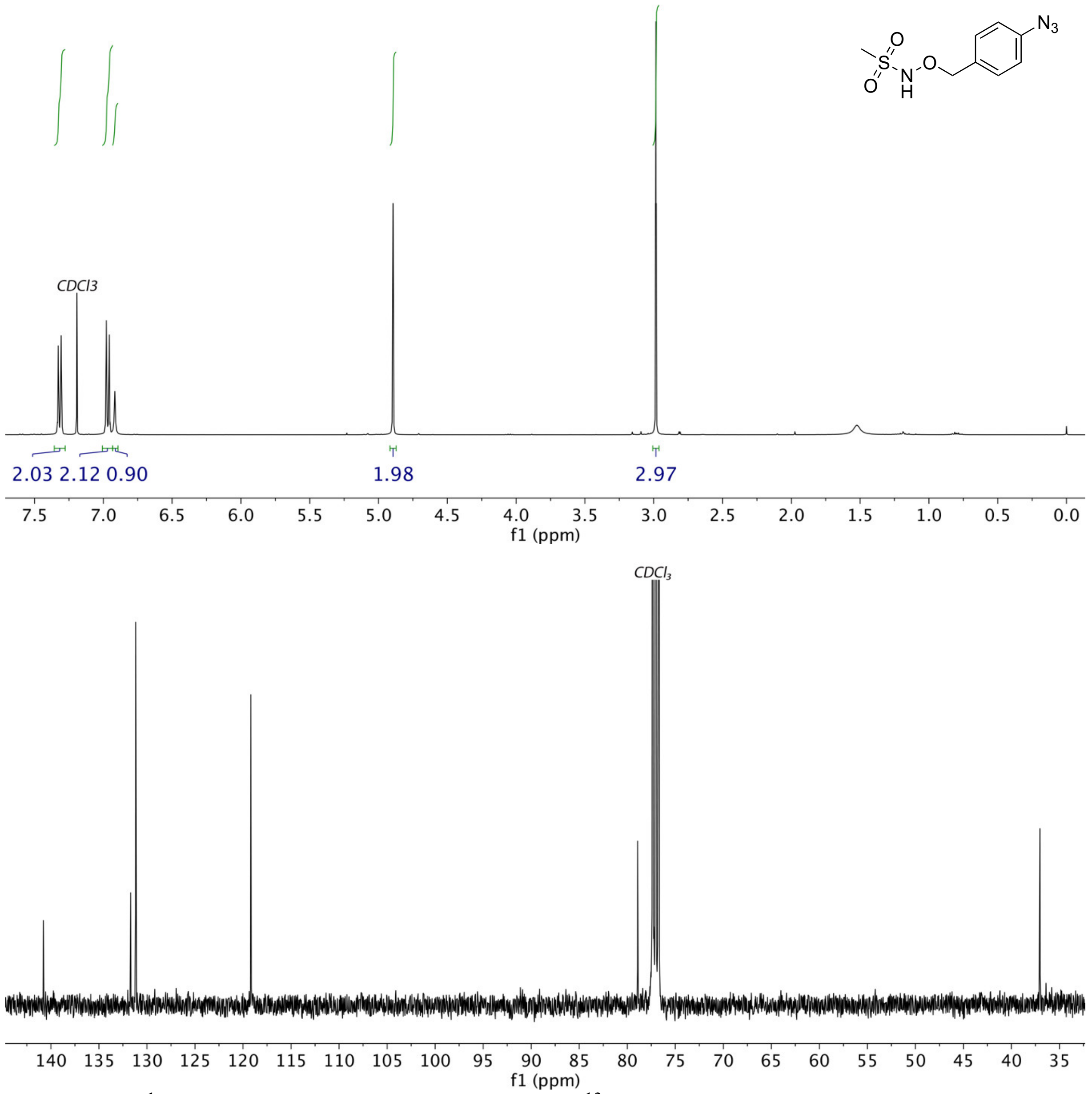

Figure S14. ^{1}H NMR (400 MHz, top) spectrum and ^{13}C NMR (101 MHz, bottom) spectrum of ***N*-((4-azidobenzyl)oxy)methanesulfonamide (2c)** in $CDCl_3$.

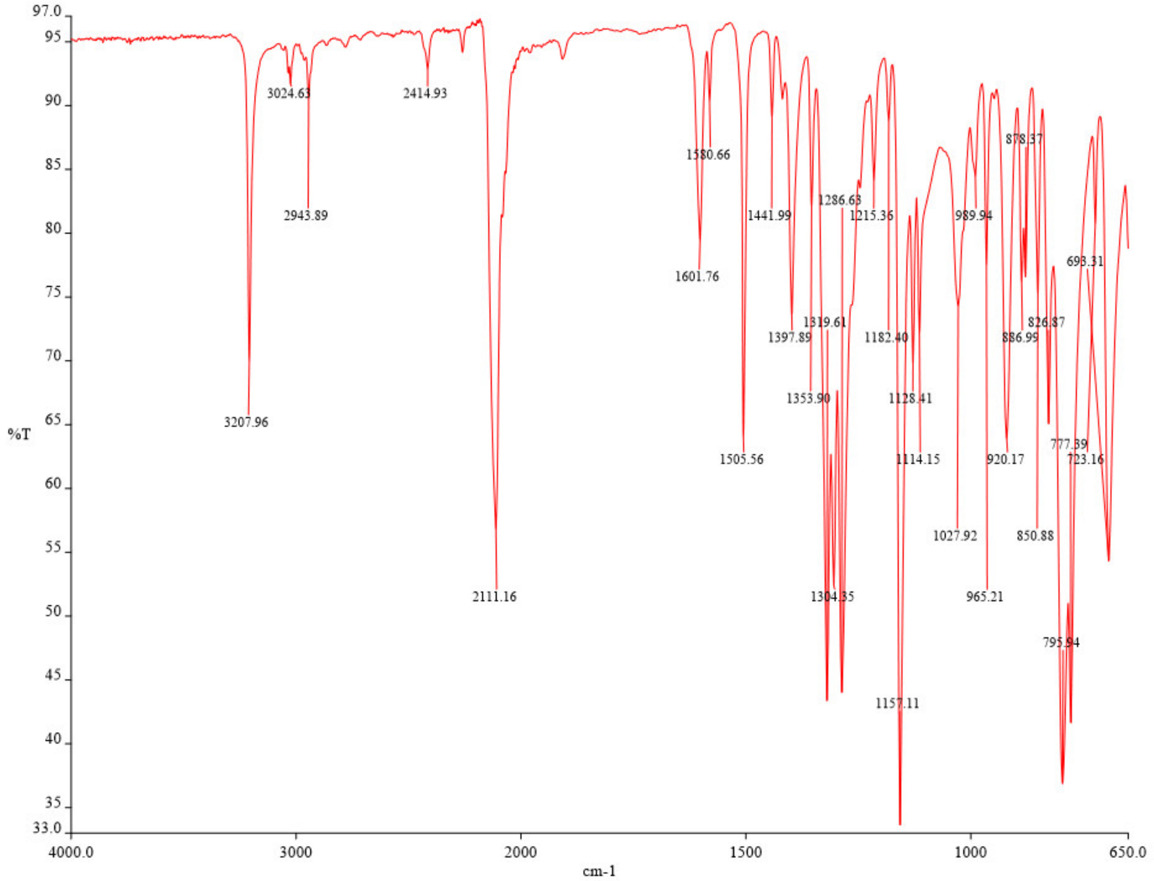

Figure S15. IR of ***N*-((4-azidobenzyl)oxy)methanesulfonamide (2c)**.

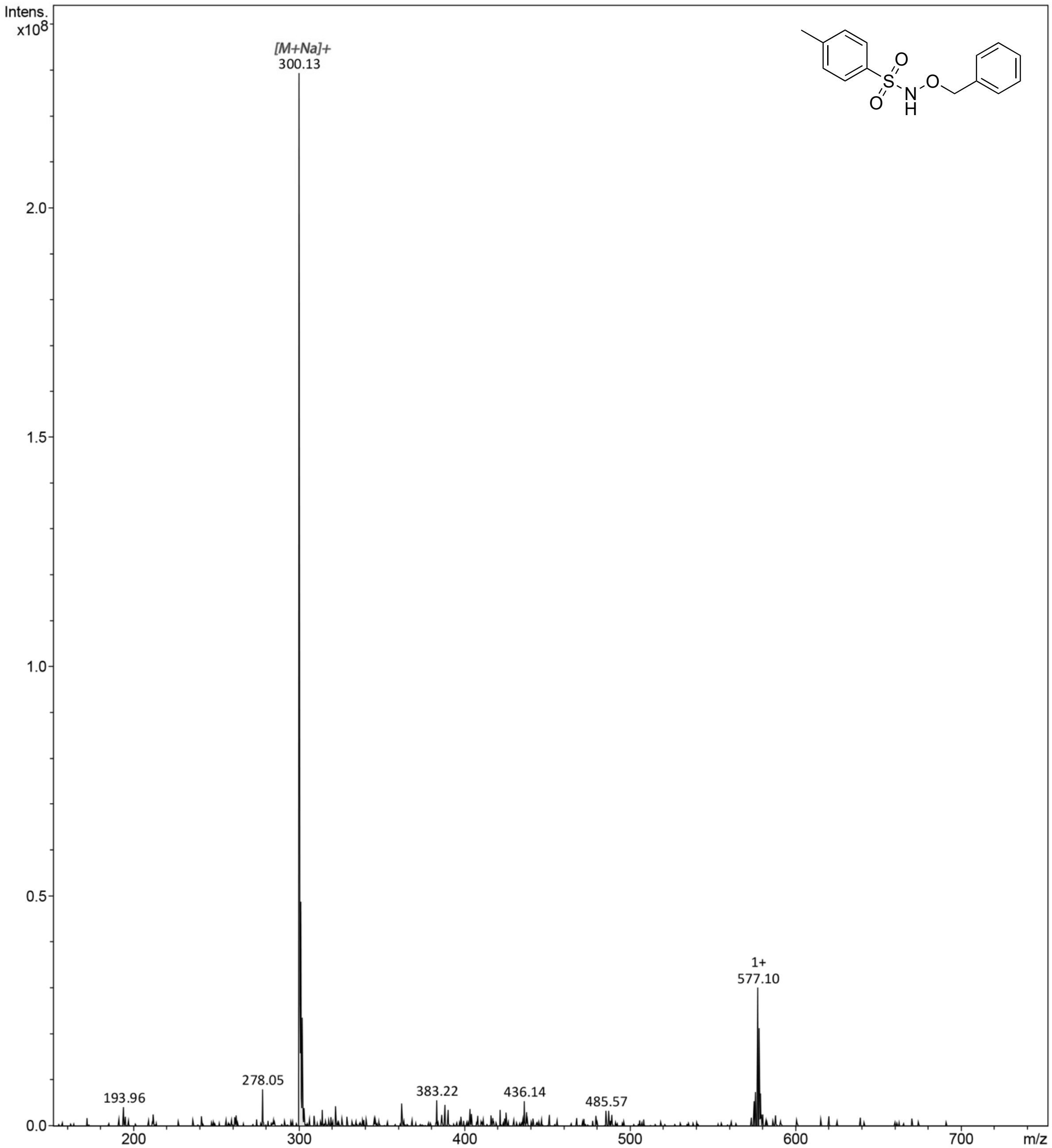

Figure S16. MS of ***N*-(benzyloxy)-4-methylbenzenesulfonamide (1a)**.

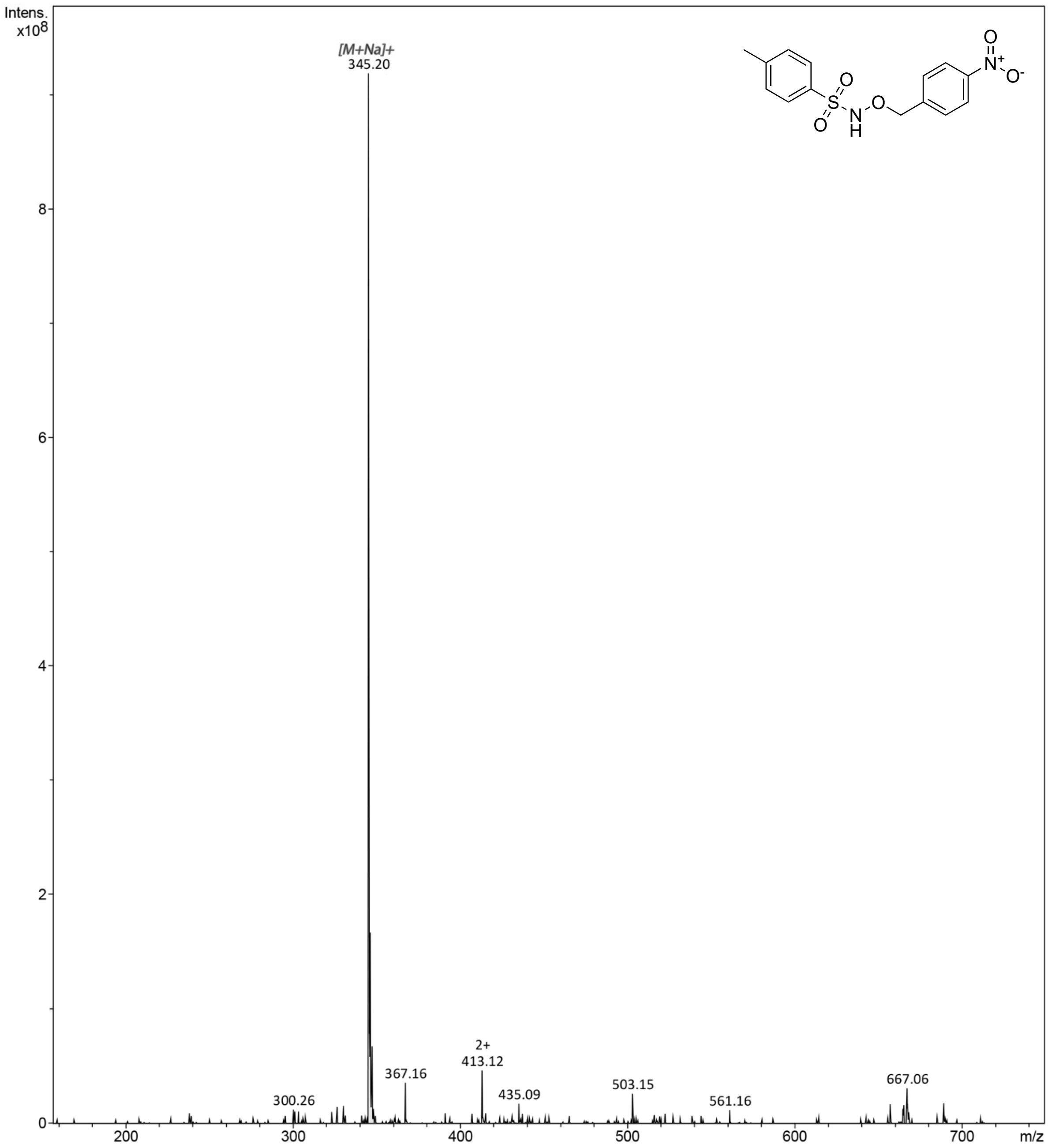

Figure S17. MS of **4-methyl-*N*-((4-nitrobenzyl)oxy)benzenesulfonamide (1b)**.

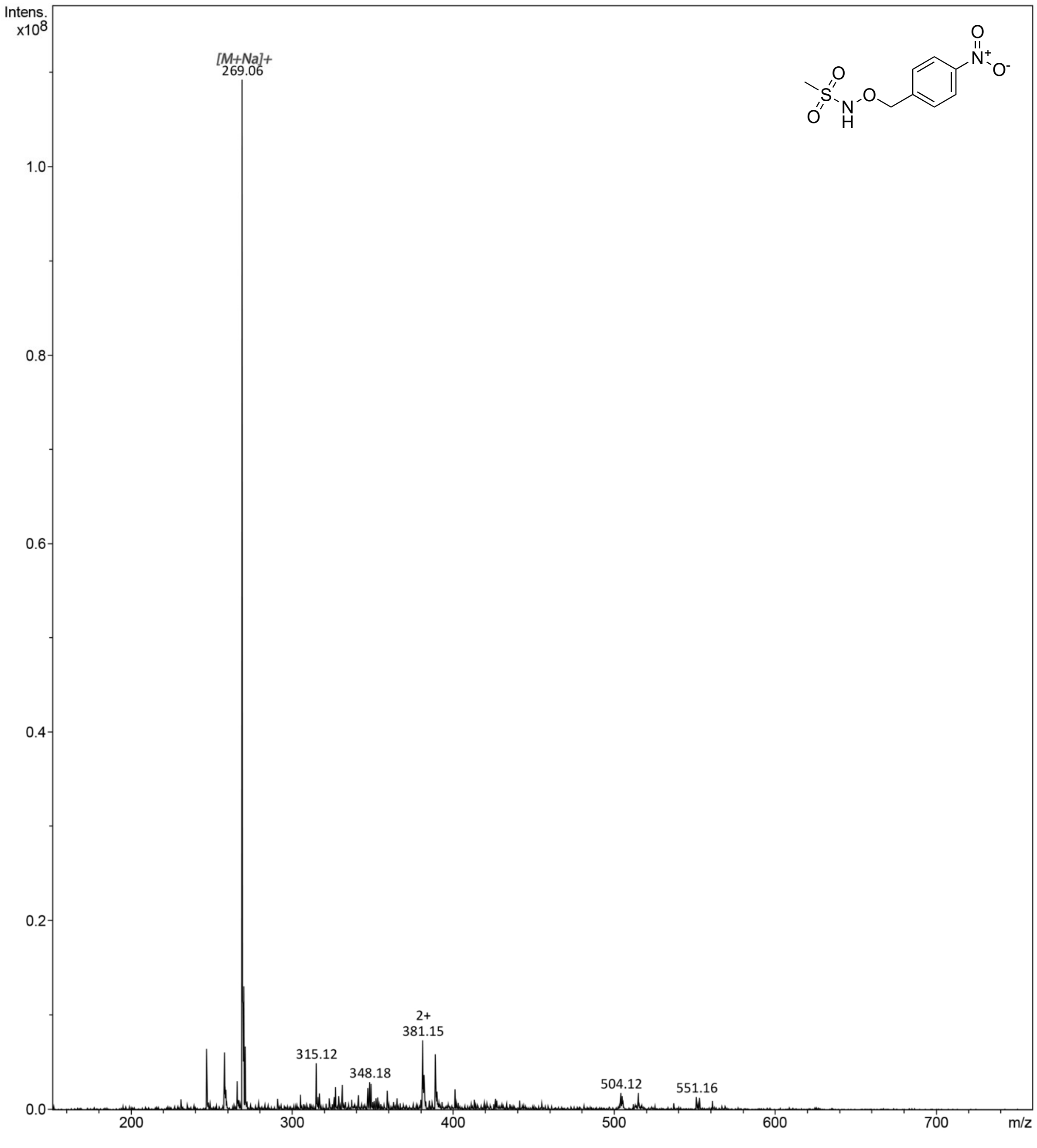

Figure S18. MS of ***N*-((4-nitrobenzyl)oxy)methanesulfonamide (2b)**.

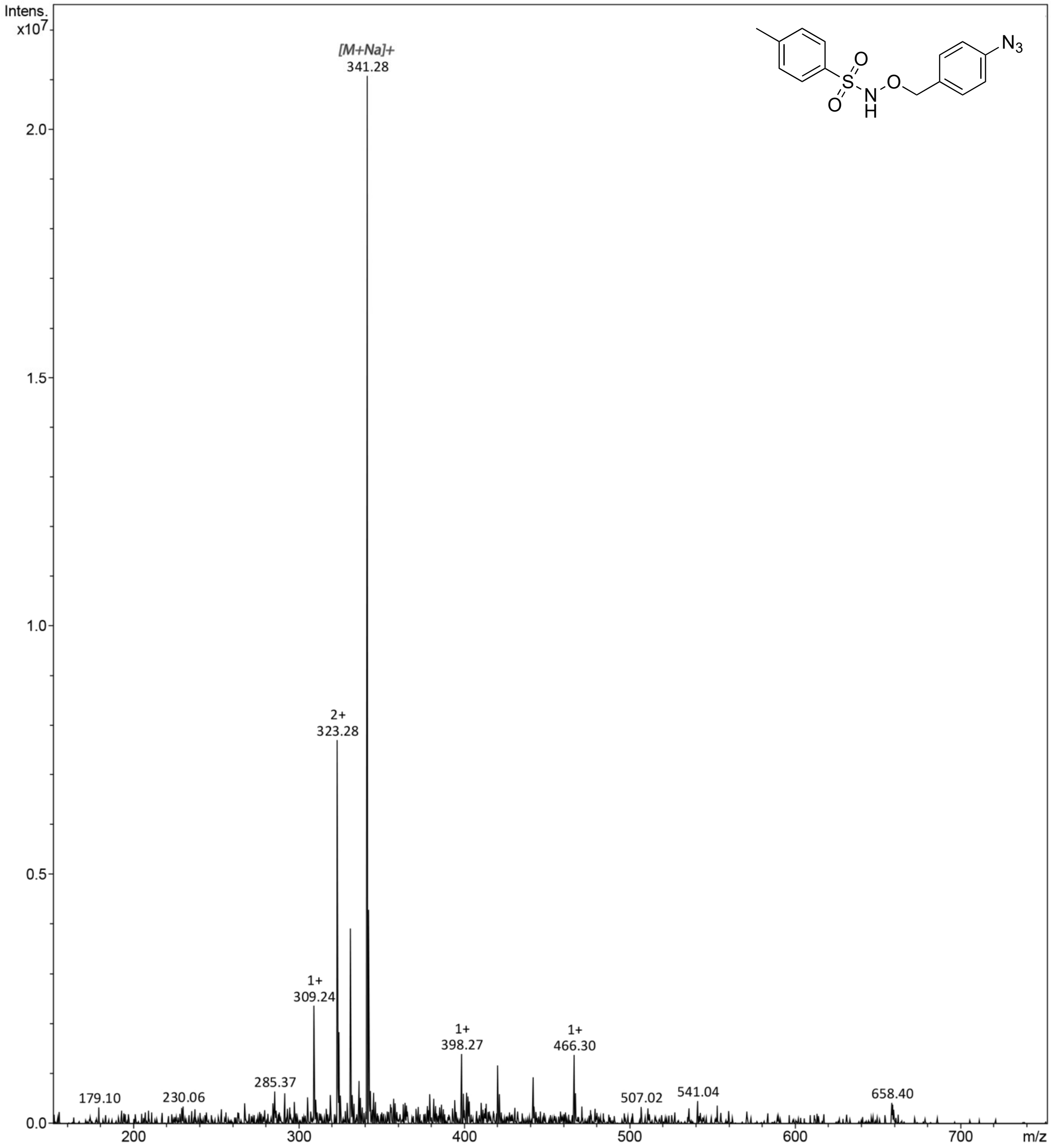

Figure S19. MS of ***N*-((4-azidobenzyl)oxy)-4-methylbenzenesulfonamide (1c)**.

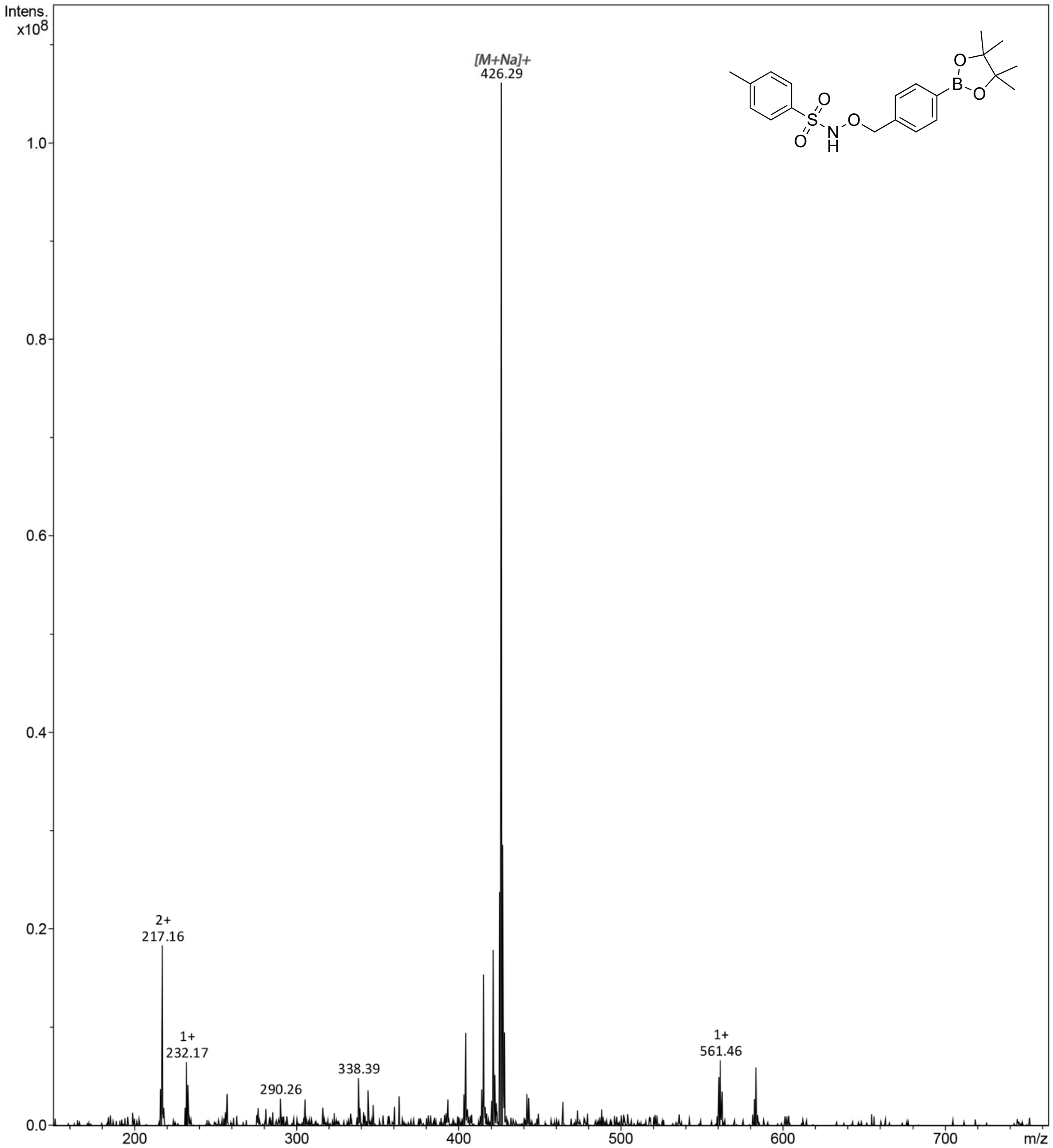

Figure S20. MS of **4-methyl-*N*-((4-(4,4,5,5-tetramethyl-1,3,2-dioxaborolan-2-yl)benzyl)oxy)benzenesulfonamide (1d)**.

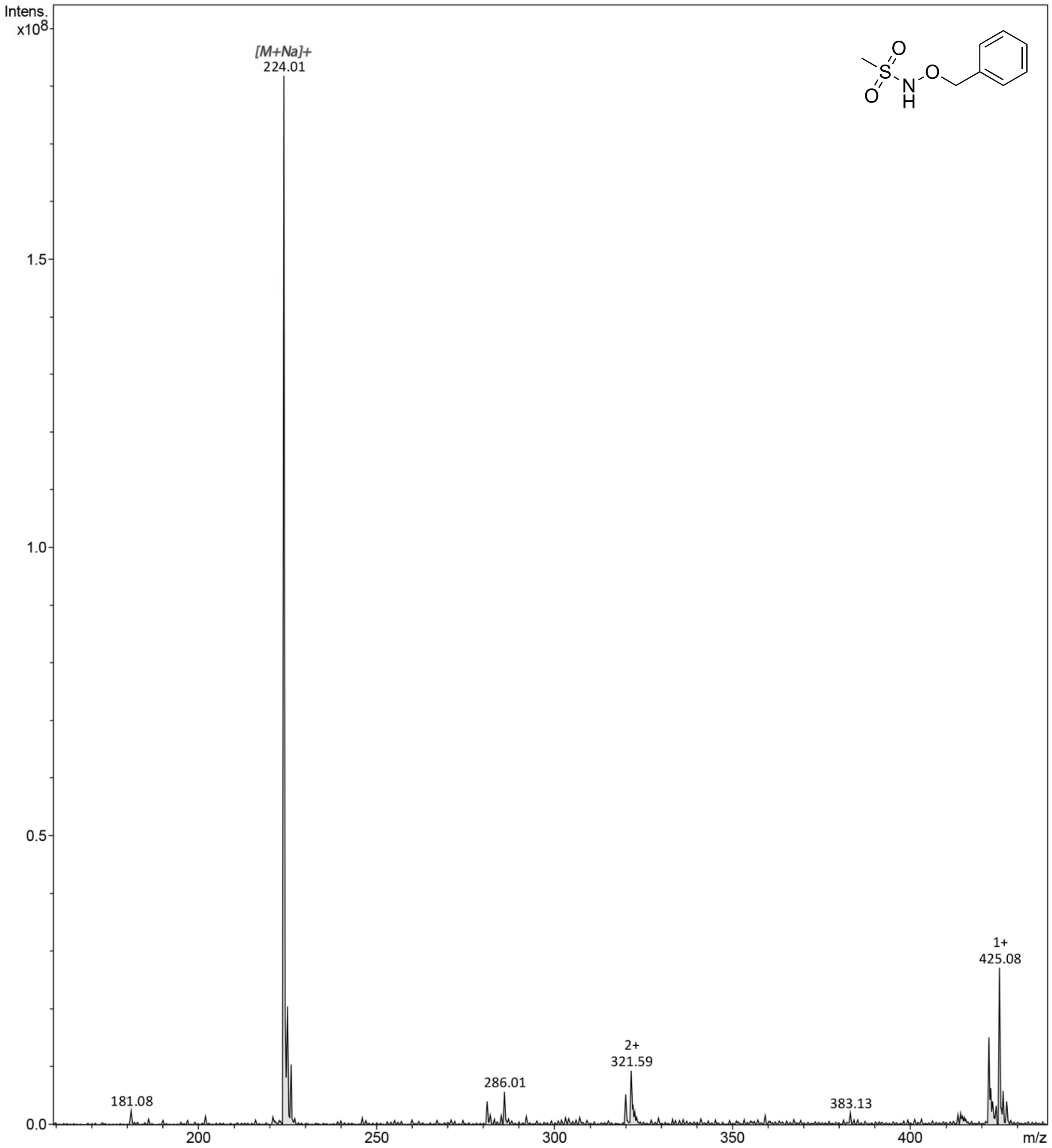

Figure S21. MS of ***N*****-(benzyloxy)methanesulfonamide (2a)**.

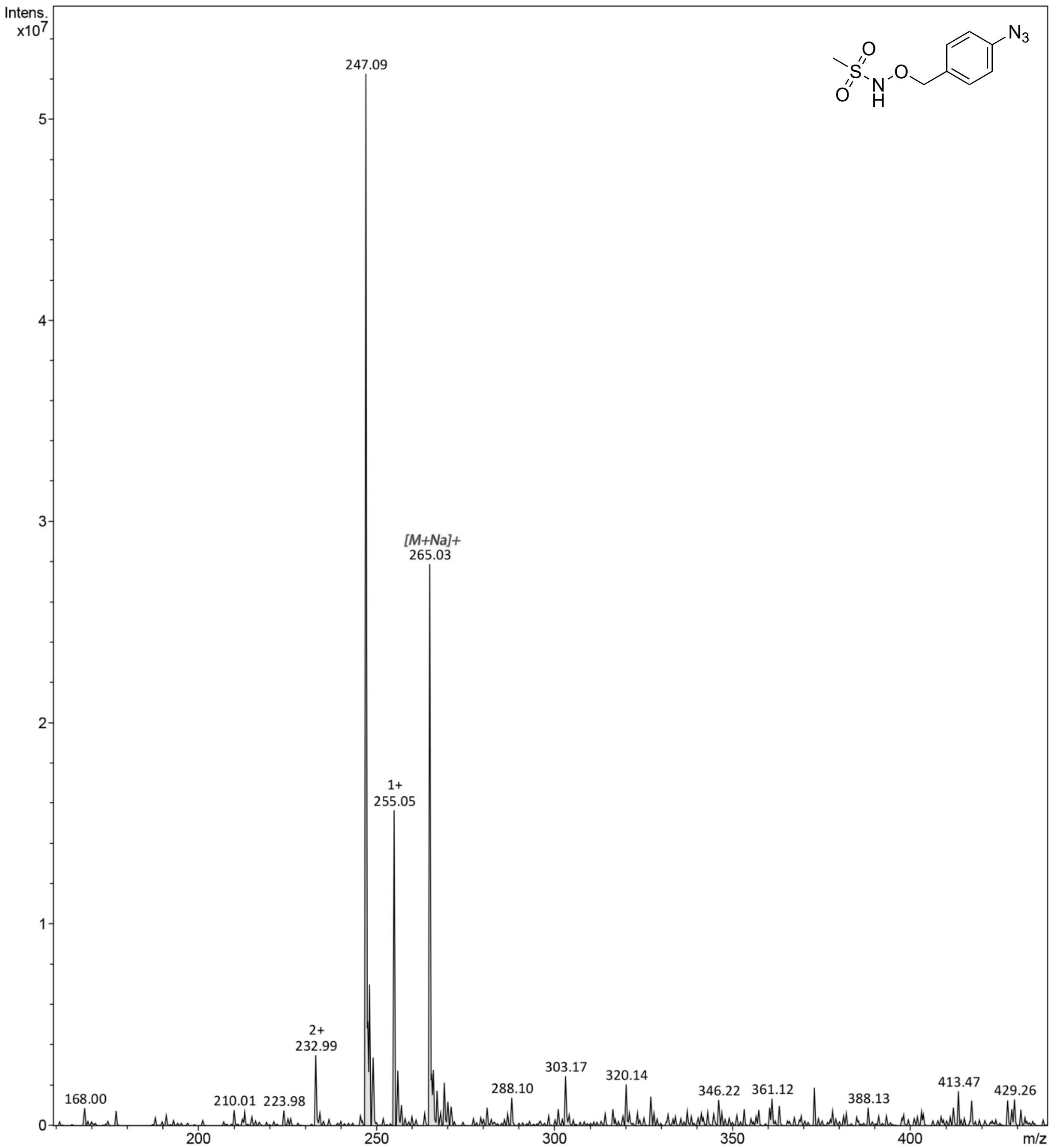

Figure S22. MS of ***N*-((4-azidobenzyl)oxy)methanesulfonamide (2c)**.
